# hsa-miR-9 controls the mobility behavior of glioblastoma cells *via* regulation of MAPK14 signaling elements

**DOI:** 10.18632/oncotarget.6687

**Published:** 2015-12-21

**Authors:** Rotem Ben-Hamo, Alona Zilberberg, Helit Cohen, Sol Efroni

**Affiliations:** ^1^ The Mina and Everard Goodman Faculty of Life Science, Bar Ilan University, Ramat-Gan, Israel

**Keywords:** MAPKAP signaling, cytoskeleton, pathways, glioblastoma, metastasis, hsa-miR9

## Abstract

**Background:**

Glioblastoma Multiforme (GBM) is the most common and lethal primary tumor of the brain. GBM is associated with one of the worst 5-year survival rates among all human cancers, despite much effort in different modes of treatment.

**Results:**

Here, we demonstrate specific GBM cancer phenotypes that are governed by modifications to the MAPAKAP network. We then demonstrate a novel regulation mode by which a set of five key factors of the MAPKAP pathway are regulated by the same microRNA, hsa-miR-9.

We demonstrate that hsa-miR-9 overexpression leads to MAPKAP signaling inhibition, partially by interfering with the MAPK14/MAPKAP3 complex. Further, hsa-miR-9 overexpression initiates re-arrangement of actin filaments, which leads us to hypothesize a mechanism for the observed phenotypic shift.

**Conclusion:**

The work presented here exposes novel microRNA features and situates hsa-miR-9 as a therapeutic target, which governs metastasis and thus determines prognosis in GBM through MAPKAP signaling.

## INTRODUCTION

Despite recent advancements in multidisciplinary therapy, GBM is still an incurable disease with a current 5-year survival rate of 9.8%. Despite therapy, once GBM progresses, the outcome is uniformly fatal, with median overall survival historically less than 30 weeks [1, 2].

The main threat and the reason for most cancer deaths are not the primary neoplasias, but secondary tumors, the metastasis. In 1926 Bailey and Cushing stated that Gliomas and Glioblastomas never metastasize outside the nervous system. Still, numerous reports on cases of extra neural metastasis have been published [3–14]. It is now known that the potential of tumor cells to metastasize depends on their interactions with the homeostatic factors that promote tumor-cell growth, survival, angiogenesis, invasion and metastasis [15]. Many of these steps depend on cell motility, which is driven by cycles of actin polymerization, cell adhesion and acto-myosin contraction [16].

MicroRNAs (miRNAs) are small, endogenous non-coding RNA molecules that control gene-expression by inhibiting translation or inducing cleavage of target mRNAs. miRNAs are involved in a wide variety of fundamental cellular processes, such as proliferation, death, differentiation, motility, invasiveness and more. miRNAs are aberrantly expressed in cancer tissue and the connection between deregulated miRNAs and the inhibition of tumor suppressor genes in cancer is well established [17]. Several studies have demonstrated a potential usefulness of miRNA-based therapy in cancer [18–20]. A good example is the use of anti-miR-21 in breast cancer, which leads to the suppression of cell growth *in vitro* and of tumor growth *in vivo* [21]. miRNAs potentially act both as therapeutic agents and as disease biomarkers, and are the subject of intense biomedical research [22].

miR-9 has been previously reported to be highly expressed in glioma cells [23–26].

In a previous work [27] using PITA [28] which is a microRNA prediction tool that scans the UTR of selected genes against all microRNAs and scores each site, we identified that hsa-miR-9 has the potential to regulate the MAPKAP network.

microRNAs (miRNAs) are generally recognized as regulating gene expression post transcriptionally by inhibiting translation or inducing target mRNA degradation. However, increasing evidences demonstrates that microRNAs can mediate mRNA translation up regulation [29]. MicroRNA-373 has been shown to induce expression of genes with complementary promoter sequences [30]. MicroRNA-466l was shown to upregulates IL-10 expression in TLR-triggered macrophages [31]. miR369-3 was found to upregulate translation of TNFα mRNA in quiescent (G0) mammalian cell lines [32]. This non-canonical regulation has been associated with non-canonical binding. In this case, YWHAZ is being regulated by miR-9 *via* 2 binding sites, both of which are non-canonical. This binding may account for the opposite trend produced by miR-9 over expression. The same trend is observed when we use anti-miR-9 and anti-con.

We hypothesized that this regulation is the outcome of targeting of several genes in that pathway, which, in turn, leads to an association with prognosis.

Our previous work identified the association between MAPKAP pathway and hsa-miR-9 using computational tools and algorithms. Here, we take the computational results and aim to validate and confirm those using experimental measures. The work presented here exposes a novel mechanism involved in this regulation. This mechanism thus suggests hsa-miR-9 as a possible therapeutic agent for treating GBM. In the work, we use *in vitro* systems to study the effects of hsa-miR-9 on MAPKAP signaling in three different GBM cell lines. We report that hsa-miR-9 alters MAPKAP signaling by directly targeting five genes within the pathway. In addition, we demonstrate that hsa-miR-9 overexpression leads to inhibition of MAPKAP signaling, partially by interfering with MAPK14/MAPKAP3 complex. This complex functions in the regulation of cytoskeleton organization. In addition, by performing phenotypic assays to characterize hsa-miR-9 effects we identified significant inhibition of both cell migration and cell invasion in the presence of the exogenous hsa-miR-9.

These findings offer a novel therapeutic target, hsa-miR-9, by demonstrating how it governs metastasis and thus determines prognosis in GBM through MAPKAP signaling.

## RESULTS

### hsa-miR-9 control patients survival and is associated with MAPKAP control mechanism

**Figure 1:**
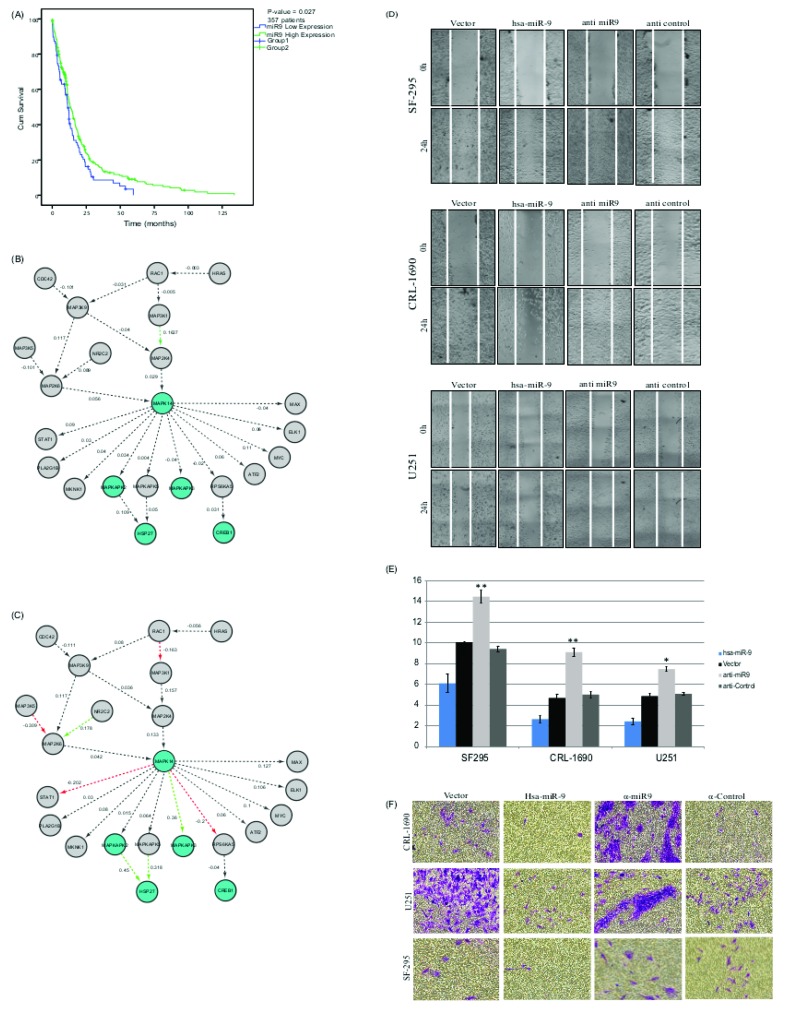
**A.** Kaplan-Meier curves generated from 357 glioblastoma patients using patient affiliation to groups parsed by expression levels of hsa-miR-9 in the TCGA dataset. Group1 (blue line, 87 patients) demonstrates lower survival rates and is affiliated with lower miR levels. Group2 (green line, 270 patients) is affiliated with higher miR levels and with better prognosis (*p*-value = 0.027). **B.** Group1 (lower survival rates and lower miR expression) *vs*. **C.** group2 (higher survival rates and higher miR levels) network. Every node represents a single protein and every edge represents a known physical interaction between the two nodes. A red-colored edge indicates a negative correlation and a green-colored edge represents a positive correlation. Every edge is marked with the calculated correlation. Nodes marked in green represents genes that are part of the original MAPKAP pathway. **D.** hsa-miR-9 reduces migration rates in GBM cell lines SF295, CRL-1690 and U251 GBM cell lines were transfected with hsa-miR-9,vector, anti-miR9 or anti-control and grown to 80-90% confluence. 48h later, transfected cells were scraped with a sterile micropipette tip to create a denuded zone (gap) of constant width. Wound gaps were monitored by Olympus CellSense at 0 and 24h after performing the scratch. **E.** Relative cellular migration area measured 24 hours post scratching, calculated using ImageJ software (NIH) for both hsa-miR9, vector, anti-miR-9 and anti-control. *P < 0.05, **P < 0.01.

Kaplan-Meier (KM) survival analysis provides a quantifiable metric to an association with disease outcome. KM analysis is often used in clinical and basic research to identify biomarkers that may improve survival rates. Here, we show that the expression levels of hsa-miR-9 stratify patients’ survival (Figure 1A). High levels are associated with significantly (*p*-value < 0.027) higher survival rates, while low expression levels are associated with lower survival rates. miR9 expression levels classification was performed using k-means clustering algorithm. K-means clustering aims to partition n observations into 2 clusters in which each observation belongs to the cluster with the nearest mean (squared Euclidean distance).

GBM disease course is such that other phenotypes (stage, pharmaceutical regiment, environmental parameters, etc.) are usually not part of the available clinical data, and disease outcome is often the only strong phenotype available. Often, molecular markers do not reveal novel mechanism but rather hide sub-clinical states which are displayed as molecular markers. To avoid such bias in our study, we set out to confirm that the stratification performed here is indeed based solely on the presented metric and is not a recapitulation of clinical variables. For this end, we performed additional analysis on possible links between the clinical measurements assessed and the groups that emerged. This analysis revealed that the classification was indeed a consequence of miR9 expression levels and not a rearrangement of well-known clinical features, demographic features or disease history. SFigure 1 shows clinical measurement distributions in the two groups according to the following features: Age, Gender, Tumor longest dimension and Histological type. The figure demonstrates that the two groups display very similar clinical features.

Here, we applied PhenoNet [33], to identify pathways and networks associated with different disease progression. PhenoNet uses two types of input data: gene expression data (RMA, RPKM, FPKM, etc.) and phenotypic information (in this case survival), and integrates these data with curated pathways and protein-protein interaction information. Comprehensive iterations across all possible pathways and subnetworks result in the identification of key pathways or subnetworks that distinguish between the two phenotypes.

PhenoNet identified a sub-network containing MAPK14 and mapkapk2/3 as highly modified version of the same mechanism in the two phenotype groups. Panels B and C in Figure 1 show the differences in both groups. Figure 1B is associated with patients in Group1 (low miR9 expression and low survival), while Figure 1C is associated with Group2 patients (high miR9 levels and high survival rates). These results indicate a possible loss of regulation within the participating genes in the group of patients with lower miR levels.

The results presented here suggest this subnetwork as a candidate molecular mechanism involved in the control of GBM disease course. When such control fails (and the correlation between the genes disappears), we see the network that characterizes the patients in the poor prognosis group.

### hsa-miR-9 regulates cell migration and invasion of glioblastoma cell lines

To see if hsa-miR-9 is associated with an oncogenic feature of cellular behavior, we measured its influence over cell migration. To do that, we used an *in-vitro* scratch assay, which is a straightforward method for measuring cell migration rates. Scratch assays measure a proxy to the ability of malignant cells to detach from their original tissue, migrate through the basal membrane, and invade other tissue. Upon creation of a new artificial gap (“scratch”) on a confluent cell monolayer, cells on the edge of the newly created gap migrate towards the “scratch” until new cell-cell contacts are established again [34–36]. To see how hsa-miR-9 over-expression influences migration, three GBM cell lines, CRL-1690, U-251 and SF-295 were grown to full confluence and transfected with hsa-miR-9, an empty vector [37], anti-miR-9, or anti-control. 48h post transfection cells were “wounded” by a sterile pipette tip. Images of the cells were taken under an inverted microscope every 30 minutes for 24 hours after the scratch; cell quantification has been analyzed using ImageJ [38]. Anti-miR-9 is miRCURY LNA miR inhibitor, which is an antisense oligonucleotides with perfect sequence complementary to its target. When introduced into cells, it sequesters the target microRNA in highly stable hetero-duplexes thereby effectively preventing the microRNA from hybridizing with its normal cellular interaction partners. Furthermore, the LNA microRNA inhibitor does not affect the microRNA endogenous expression levels; by binding to the microRNA the LNA only prevent the miR from binding to other targets.

This analysis showed an average decrease of 2-fold in migration rate in the presence of hsa-miR-9, compared to vector, and an average decrease of 3 fold compared to anti-miR (Figure 1D, 1E).

In addition, an invasion assay was performed in order to examine the effect of the miR on the cells migration ability (Figure 1F, 1G). As can be seen from the figure, the cells’ ability to invade the ECM is reduced in the presence of hsa-miR-9, compared to the control. In addition, the cells’ invasion ability is recovered in the presence of the anti-miR. These results highlight the strong effect of miR-9 on glioblastoma cells’ mobility.

### hsa-miR-9 regulates the MAPKAP signaling pathway by regulating six pathway members

**Figure 2:**
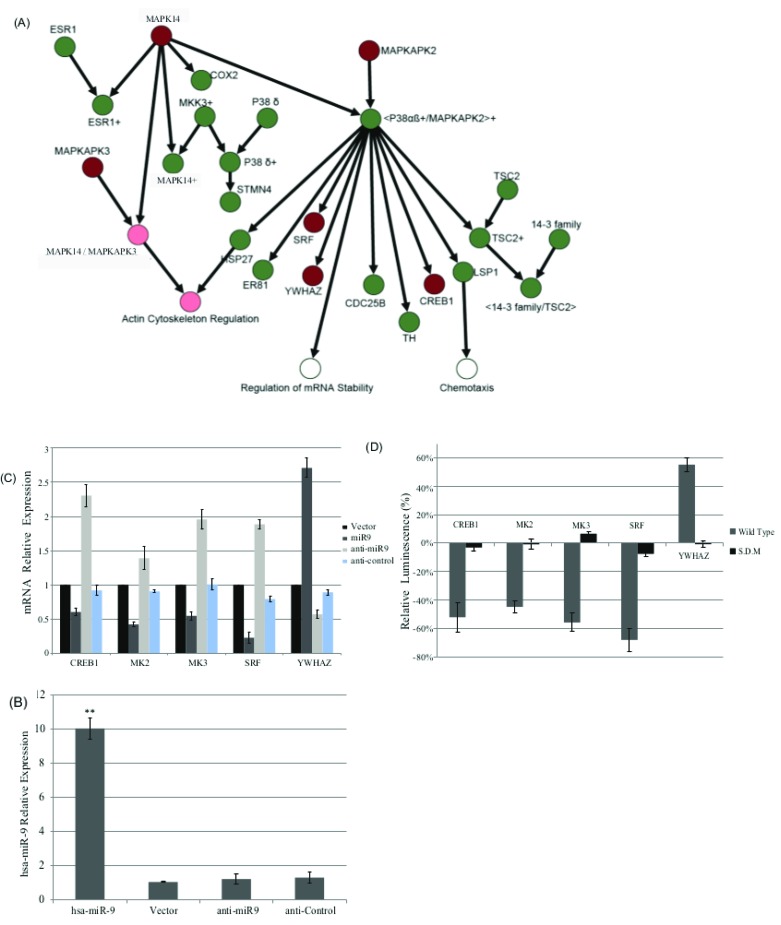
**A.** MAPKAP signaling pathway. Genes highlighted in red are in the MAPKAP network, both computationally and experimentally, to be targeted by hsa-miR-9. Nodes highlighted in light red are the processes found to be interfered by the effect of the miR on the pathway. **B.** hsa-miR-9 targets MAPKAP pathway in a direct manner.CRL1690 cells were transfected with hsa-miR9, vector, anti-miR9 or anti-control. 72h later hsa-miR9 levels were measured by qRT-PCR and normalized to RNU6B endogenous control. **C.** Five canonical MAPKAP pathway partners were measured for their mRNA levels upon transfection with vector, hsa-miR9, anti-miR9 or anti-control. Results demonstrated reduction in mRNA transcripts upon miR9 transfection, in four out of five cases. *P < 0.05, **P < 0.01. **D.** CRL1690 cells were co-transfected with hsa-miR9 or vector, and the 3’ UTR of the indicated genes (canonical MAPKAP pathway partners) fused to Firefly luciferase and Renilla Luciferase or their corresponding mutant 3’UTR promoters. 72h post transfection, cell lysates were measured for their luminescence normalized to Renilla. Relative Luciferase values represent the ratio miR9: vector for each detected promoter. Upon miR9 transfection, most of the genes exhibited down regulation in their promoter activity.

In previous work [27], we identified a negative correlation between the expression levels of hsa-miR-9 and MAPKAP activity. We also identified a possible hsa-miR-9 target site on five genes within this pathway (CREB, MAPKAPK2 (MK2), MAPKAPK3 (MK3), SRF and YWHAZ) (Figure 2A).

To understand the specific mode of regulation used by hsa-miR-9, we measured (qRT-PCR) mRNA levels of these five genes of the MAPKAP cascade, by using the following strategies:

CRL-1690 cells transfected with hsa-miR-9, anti hsa-miR-9 and anti-control or an empty vector, were measured for the mRNA levels of five canonical MAPKAP pathway partners

Results demonstrated reduction in mRNA transcripts upon hsa-miR-9 transfection, for all genes, excluding YWHAZ (Figure 2C).

A specificity assay was designed to evaluate the native promoter activity for each listed gene upon hsa-miR-9 over expression.CRL-1690 cells were co-transfected with hsa-miR-9 or vector and the 3’UTR promoter of the indicated genes: CREB, MAPKAPK2 (MK2), MAPKAPK3 (MK3), SRF and YWHAZ fused to Firefly Luciferase and Renilla Luciferase expression vector or their respected mutated version of each 3’UTR promoters .72h post transfection, cell lysates were measured for their luminescence and normalized to Renilla. Relative Luciferase values represent the ratio hsa-miR-9: vector for each detected promoter. Upon introduction of hsa-miR-9, four out of the five genes demonstrated reduction of their promoter activity, while their corresponding mutant 3’UTR demonstrated very low luminescence or not at all (Figure 2D). Figure 2D shows how all five genes are directly targeted by hsa-miR-9: four genes exhibited an inhibition trend while one gene, YWHAZ, upregulated upon hsa-miR-9.

Furthermore, the results presented here highlight MAPK14/mapkapk2/mapkapk3 and their interaction with hsa-miR-9 as a possible molecular mechanism involved in the control of GBM progression.

### hsa-miR-9 interferes with the MAPK14/MAPKAP3 complex production by down- regulating both MAPK14 and MAPKAP3 levels

MK2 and MK3 are essential cell migration factors [39], which are regulated by phosphorylation mediated by MAPK14, MAPKα and MAPKβ [40]. Moreover, MAPK14/MAPK activity is required both for actin polymerization and for its dynamics [41] and is known to modulate actin cytoskeleton in endothelial cells [42, 43].

To study the association between hsa-miR-9 over expression and the observed reduction in the invasion and migration of the cancer cells, as well as the observed association with prognosis across cohort of patients, we conducted a western blot assay. We detected whether hsa-miR-9 interferes with the MAPK14 /MAPKAP3 complex formation by interfering with their expression levels. MAPKAP signaling through MAPK14/MAPKAP3 junction results in actin cytoskeletal regulation (Figure 2A) and could thus account for the observed reduction in migration and in invasion.

**Figure 3:**
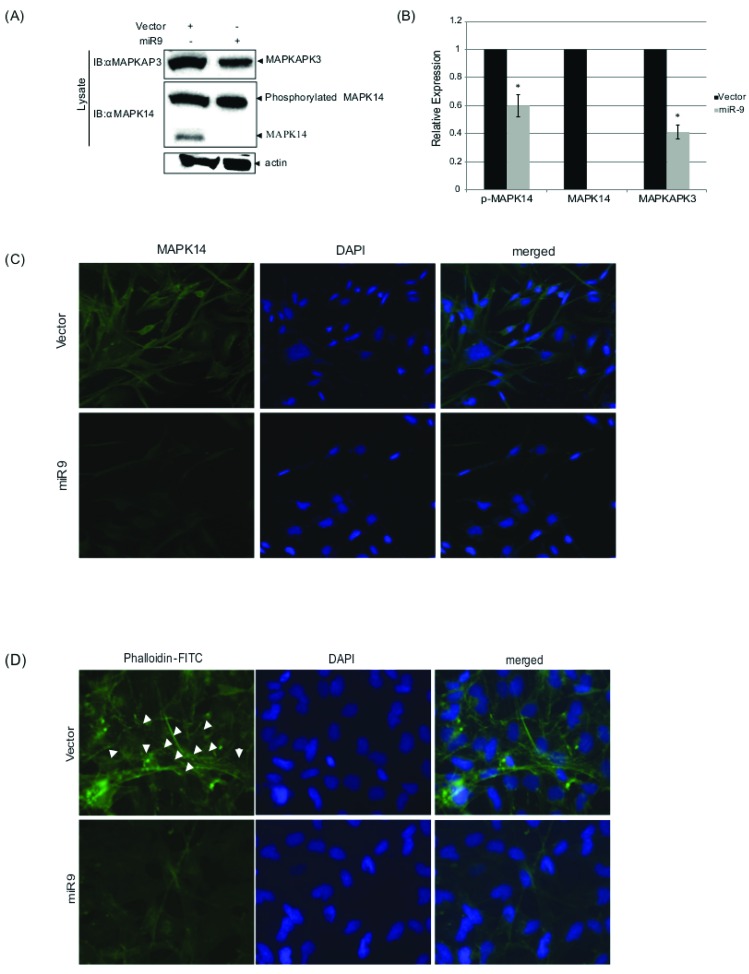
hsa-miR-9 decrease MAPK14 and MAPKAP3 protein levels CRL-1690 cells were transfected with hsa-miR9 or vector. 48h later, cells lysates were loaded on SDS-PAGE gel as compared to their corresponding input controls which were measured for actin as a loading control. Proteins were transfer to a nitrocellulose membrane and blotted to the indicated antibodies. Actin measurement served as a loading control. GFP protein levels were quantified compared to Actin. *P < 0.05. **C.** hsa-miR-9 decreases MAPK14 expression levels. CRL-1690 cells grown on cover slips were transfected with hsa-miR9 or vector. 48h later, transfected cells were fixed and permeabilized in the presence of weak detergent, followed by incubation with 5μg/ml of anti-MAPK14 (ab-9352) over-night at 4°C and then were introduced with secondary Alexa Fluor 488 Goat Anti-Rabbit IgG (H+L) Antibody for additional 45 min at Room temperature. 4-6’ diamidino-2 phenylindole (DAPI, Sigma) was used to stain cell nuclei. AxioimagerZ1 microscope monitored the MAPK14 patterns obtained upon vector or miR9 transfection **D.** hsa-miR-9 induces re-organization of Actin filaments *via* interfering with MAPK14/MAPKAP3 complex CRL-1690 cells grown on cover slips were transfected with hsa-miR9 or vector. 48h later, transfected cells were fixed and permeabilized in the presence of weak detergent, followed by incubation with 50mg/ml Phalloidin - FITC for 40 min. DAPI was used to stain nuclear localization. AxioimagerZ1 microscope monitored the actin filament patterns obtained upon vector or miR9 transfection. White arrows point normal organization of Actin filaments.

Results, shown in Figure 3A, 3B, demonstrate that upon hsa-miR-9 overexpression, endogenous levels of both MAPK14 and MAPKAP3 decrease.

As Figure 3A shows, protein levels of MAPKAP3 decrease in the presence of hsa-miR-9. Surprisingly, the phosphorylated form of MAPK14 shows no significant changes upon introduction of hsa-miR-9 (band size 37kDa), while the un-phosphorylated form of MAPK14 was at undetectable levels (band size 25kDa), even though the 3’UTR of MAPK14 does not contain any hsa-miR-9 binding sites.

Further, CRL1690 cells, which were transfected with hsa-miR-9, demonstrated significantly lower levels of endogenous MAPK14, compared to CRL1690 cells, which were transfected with vector alone (Figure 3C). These results strengthen the demonstrated western blot results which showed a decrease in MAPK14 levels, induced by over expression of hsa-miR-9.

Together, these results indicate that the hsa-miR-9 induced decrease in migration and invasion of GBM cells, is directly mediated through MAPKAP signaling.

### hsa-miR-9 interferences with MAPK14/MAPKAP3 complex induces F-actin re-organization

During stress, an increased stability of the actin cytoskeleton is mediated by activation of MAPK14 [40]. Upon hsa-miR-9 expression, MAPK14 activation is blocked by a reduction in the non-active form of MAPK14, as demonstrated in Figure 3A, 3B and 3C. Initiation of MAPK14/MAPKAP3 complex mediates cytoskeleton organization (as was previously reported). Thus, a decrease in this complex formation induced by hsa-miR-9 may result in a decline in cell motility, through interruption with the actin cytoskeleton.

CRL1690 cells were seeded on glass cover slips and transfected with hsa-miR-9 or vector. 48h post transfection cells were fixed, permeabilized and introduced to phalloidin-FITC to stain for F-actin. DAPI was used to stain nuclear localization (Figure 3D). hsa-miR-9 thus controls a unique F-actin pattern. This display is dominated by interference with the MAPK14/MAPKAP3 complex, as demonstrated above.

## DISCUSSION

miRNAs, a class of small noncoding RNAs, have emerged as important devices of post transcriptional regulation. A large, and growing, number of reports present the role of miRNAs in cancer [44–47]. In addition to the known and well-established role of miRNAs as inhibitors of gene expression, several works have demonstrated miRNAs ability to up-regulate gene expression [30–32, 48].

As every miRNA can potentially target hundreds of different transcripts simultaneously, in principle, the regulation of a single miRNA may lead to regulation over en entire pathway, by interactions with multiple targets within the pathway. While the influence of miRNAs over pathway cascades has been demonstrated [49–53], reports are showing the cascade effect followed by down-regulation of one key member of the pathway, and not by intervention through multiple targets.

Mitogen-activated protein kinase (MAPK) cascades have been shown to play a key role in transduction extracellular signals to cellular responses [54, 55]. Furthermore, previous reports have shown the role of the additional targeted genes in this pathway (SRF, CREB1, YWHAZ, and MAPKAPK2) in actin cytoskeleton reorganization. SRF for an example controls growth factor regulated immediate-early genes such as cytoskeletal actin [56, 57], YWHAZ mediate elongation in monocytic cells *via* Rac-1 mediated actin cytoskeleton reorganization [58]. The major role of MAPKAPK2 and HSP27 in regulation of actin is well established [59, 60]. However, the effect we demonstrate here has thus not been reported before and presents a novel regulatory mechanism.

In the work presented here we demonstrate a novel and unique miRNA feature, by which a single miRNA targets an entire pathway, through the regulation of a subset of genes of the same cascade, ultimately leading to the phenotype of attenuation in cell migration and invasion.

As the paper shows, interfering with gene expression in the MAPKAP signaling pathway, leads to a reduction of MAPK14/MAPKAPK3 complex formation. This complex is known to mediate cell cytoskeleton re-organization. We hypothesize that this disruption of the MAPK14-MAPKAP3 complex homeostatic function accounts for the decrease in cell migration, cell invasion and ultimately of the clinical phenotype associated with hsa-miR-9 levels, and especially of the clinical phenotype observed in conjunction with the MAPKAP pathway activity [27].

These previously reported results, which demonstrated higher survival rates in patients with a negative miRNA-pathway association, highlight this critical miRNA-pathway mechanism. In the work presented here, we expanded our results and identified the specific players, their molecular interactions within the MAPKAP signaling pathway, their regulation by hsa-miR-9 and their control over the observed phenotype, using the presented results and by combining computational and experimental work we can catalyze targeted treatment, facilitate prognosis through network biomarkers and offer a novel perspective into hidden disease heterogeneity. This is the first evidence of a single microRNA directly targeting a subset of genes at the same pathway.

## MATERIALS AND METHODS

### Cell lines and transfection

ATCC CRL1690 (T98G), U251 and SF295 Glioblastoma cell lines were used for all experiments. Cells were maintained according to the supplier's instructions. Briefly, cells were maintained in MEM Eagle's medium supplemented with 10% FCS and 100U/ml penicillin/streptomycin at 37°C in a humidified 5% CO_2_ atmosphere. Transfections were performed by the DNA transfection reagents jet PEI (Polyplus Transfection), following the manufacturers protocols.

### qRT-PCR

Total RNA was prepared with Trizol reagent according to manufacturer's protocol. miRNA was subjected to reverse transcription using the Taqman microRNA reverse transcription kit (Applied Biosystems) as previously described [61]. Briefly, real-time PCR was run on the Applied Biosystems 7900HT machine (Applied Biosystems, Life technologies Co.GI, NY, USA). Relative expression levels of miR9 were normalized to U6 snRNA. U6 snRNA was used as the endogenous control for all experiments. At least three biological replicates were included for each condition. For target gene expression, total RNA were subjected to Syber FAST ABI Prism qPCR Kit (KapaBiosystems Inc. Woburn, MA, USA) Reactions were run on 7900HT Real Time PCR. Relative expression levels of each gene were normalized to actin endogenous control.

### Anti-miRNA-9

miRCURY LNA™ miR Inhibitors are antisense oligonucleotides with perfect sequence complementary to their target. When introduced into cells, they sequester their target miR in highly stable hetero-duplexes thereby effectively preventing the miR from hybridizing with its normal cellular interaction partners. Further, the LNA microRNA inhibitor does not affect the miR endogenous expression levels; by binding the miR, the LNA only prevents the miR from binding to other targets.

### Vector and hsa-miR-9

hsa-miR-9 and its corresponding control (“Vector”) were both a generous gift from Reuven Agami. Comprehensive explanation regarding the vectors construction can be found in [37].

### Plasmid constructs

The 3’-UTRs of the following genes: CREB1, MAPKAPK2, MAPKAPK3, SRF, and YWHAZ were amplified on a cDNA template generated from CRL-1690 cells, purified and cloned into psi-CHECK2 dual luciferase reporter plasmid downstream to the Renilla luciferase coding region. All generated constructs were verified by sequencing.

### Site directed mutagenesis manipulation

We introduced a point mutation in the 3’-UTRs of the following genes: CREB1, MAPKAPK2, MAPKAPK3, SRF and YWHAZ using an appropriate primers set and the QuickChange II Site-Directed Mutagenesis Kit (Agilent Technologies) on the psi-CHECK2 dual luciferase reporter plasmid of each gene, respectively.

### Dual luciferase reporter assay

Luciferase reporter assays were performed using the psiCHECK2 vector. 1 × 10^3^ cells were seeded per 96-well plates and cotransfected with 0.7 μg miR-9 or miR-vector along with 0.3 μg of psiCHECK2 construct (representing the 3’ UTR of the 5 indicated genes). 48h post transfection, the cells were harvested and subjected to the Dual-Luciferase Reporter Assay system (Promega): Renilla luciferase activity was normalized to firefly luciferase activity.

### Scratch assay

The motility of CRL-1690 cell line was assessed by a scratch wound healing assay which designed to measure the expansion of a cell into a sterile zone. Cells were seeded into 6-well plate and transfected with miR-9 or miR-vector. 24 hours later, we extracted the medium and created a denuded zone of constant width (wound) at the middle of the confluent cell monolayer, using a sterile micropipette tip Images were taken at 0 and 24 h intervals post scratching. ImageJ was used to determine the migration rate by measuring the distance between the edges. The experiment was repeated three times.

### Invasion assay

*In vitro* cell invasion was assayed using an extracellular matrix (ECM) invasion assay kit (ECMatrix Cell Invasion Assay (catalog # ECM550, Millipore, Billerica, MA) according to the manufacturer's protocols. Cells were seeded into 6-well plate and transfected with miR-9 or miR-vector. 24 hours later 3×10^5^ transfected cells were suspended in 300μl of serum-free media and plated on the top of an ECM-coated membrane insert for 2 h incubation. The insert was then incubated for 48 h in serum-containing media. The non-invading cells were gently removed from the upper chamber with cotton-tipper swabs, and filters were incubated in staining solution for 20 min, rinsed several times in water and air dried. Cells that invaded to the underside of the ECM membrane were quantified using ImageJ (NIH, Bethesda, MD) software. The experiments were repeated three times.

### Western blot analysis

Cells were seeded into 10cm plates and transfected with miR-9 or miR-vector. Forty-eight h following transfection, cells were washed with PBS and solubilized in M2 lysis buffer (100 mM NaCl, 50 mM Tris, pH7.5, 1% Triton X-100, 2 mM EDTA) containing protease inhibitor cocktail (Sigma). Extracts were clarified by centrifugation at 12,000 x *g* for 15 min at 4°C. Next, proteins were transferred to nitrocellulose and blocked with 5% low fat milk.

Membrane was incubated with anti- MAPK14 and anti-MAPKAP3 specific primary antibodies, washed with PBS containing 0.001% Tween-20 (PBST) and incubated with the appropriate horseradish peroxidase-conjugated secondary antibodies. After washing in PBST, membranes were subjected to enhanced chemiluminescence (ECL) detection analysis.

### Immunofluorescence

CRL1690 cells were grown on coverslips in a 6-well plate and transfected with miR-9 or miR-vector. 48 hours later cells were fixed for 20 min in PBS containing 4% paraformaldehyde, washed 3 times with PBS, and permeabilized at the presence of 0.1% Triton X-100 for 10 min. Follow, cells were blocked with 1% bovine serum albumin for 1 hour. Subsequently, cells were incubated at room temperature with 50μg/ml of Phalloidin-FITC for 40 min or alternatively cells were incubated with 5μg/ml of anti-MAPK14 (ab-9352), over-night at 4C and then were introduced with secondary Alexa Fluor 488 Goat Anti-Rabbit IgG (H+L) Antibody for additional 45 min. 4-6’ diamidino-2 phenylindole (DAPI, Sigma) was used to stain cell nuclei. Cells were visualized by AxioImager Microscope.

## SUPPLEMENTARY MATERIAL FIGURE


