# Supplementary material for: hsa-miR-9 controls the mobility behavior of glioblastoma cells via regulation of MAPK14 signaling elements
Source: Oncotarget. 2015 Dec 21;7(17):23170–81. doi: 10.18632/oncotarget.6687 (PMC5029618; doi:10.18632/oncotarget.6687)
Supplement: Supplementary file 1 [file oncotarget-07-23170-s001.pdf]

hsa-miR-9 controls the mobility behavior of Glioblastoma cells *via* regulation of MAPK14 signaling elements

Supplementary Material

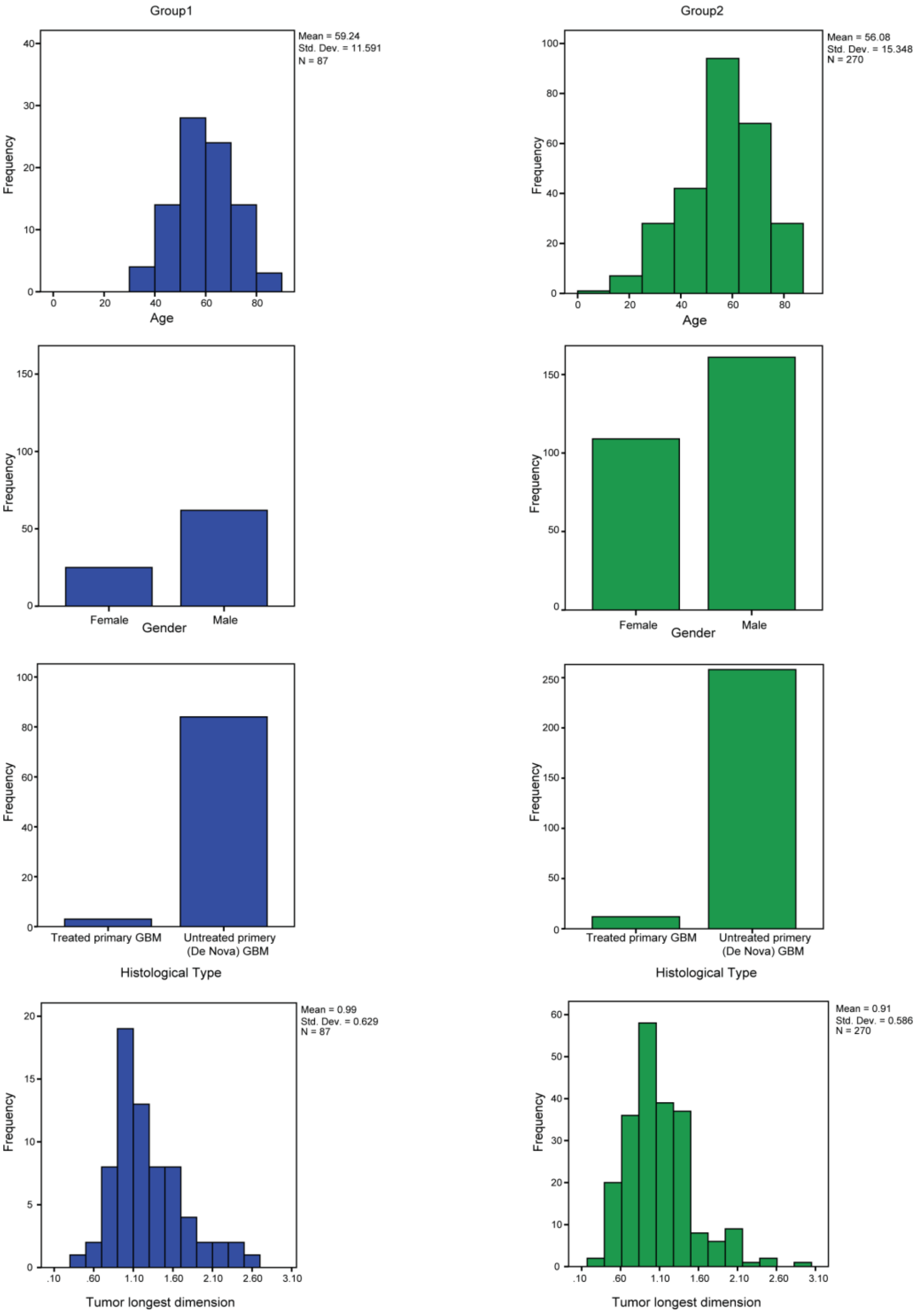

SFigure 1: Clinical measurement distributions in the two groups according to the following features: Age, Gender, Tumor longest dimension and Histological type. The figure demonstrates that the two groups display very similar clinical features.
